# The first familial *NSD2* cases with a novel variant in a Chinese father and daughter with atypical WHS facial features and a 7.5-year follow-up of growth hormone therapy

**DOI:** 10.1186/s12920-020-00831-9

**Published:** 2020-12-04

**Authors:** Xuyun Hu, Di Wu, Yuchuan Li, Liya Wei, Xiaoqiao Li, Miao Qin, Hongdou Li, Mengting Li, Shaoke Chen, Chunxiu Gong, Yiping Shen

**Affiliations:** 1 Beijing Key Laboratory for Genetics of Birth Defects, Beijing Pediatric Research Institute; MOE Key Laboratory of Major Diseases in Children, Beijing Children’s Hospital, Capital Medical University, National Center for Children’s Health, Beijing, 100045 China; 2 Genetics and Birth Defects Control Center, Beijing Children’s Hospital, Capital Medical University, National Center for Children’s Health, Beijing, 100045 China; 3 Department of Endocrinology, Genetics and Metabolism, Beijing Children’s Hospital, Capital Medical University, National Center for Children’s Health, No.56 South Lishi Road, Xicheng District, Beijing, 100045 PR China; 4 Obstetrics Gynecology Hospital, The Institute of Reproduction and Developmental Biology, Fudan University, Shanghai, 200011 China; 5 Genetic and Metabolic Central Laboratory, Maternal and Child Health Hospital of Guangxi Zhuang Autonomous Region, Nanning, 530023 Guangxi China; 6 The second affiliated hospital of Guangxi Medical University, Nanning, 530000 Guangxi China; 7 Shanghai Children’s Medical Center, Shanghai Jiao Tong University School of Medicine, Shanghai, 200011 China; 8 Division of Genetics and Genomics, Boston Children’s Hospital, Harvard Medical School, 300 Longwood Ave, Boston, MA 02115 USA

**Keywords:** Wolf-Hirschhorn syndrome, *NSD2* gene, Growth hormone therapy, Facial dysmorphism, Intellectual disability

## Abstract

**Background:**

Wolf-Hirschhorn syndrome is a well-characterized genomic disorder caused by 4p16.3 deletions. Wolf-Hirschhorn syndrome patients exhibit characteristic facial dysmorphism, growth retardation, developmental delay, intellectual disability and seizure disorders. Recently, *NSD2* gene located within the 165 kb Wolf-Hirschhorn syndrome critical region was identified as the key causal gene responsible for most if not all phenotypes of Wolf-Hirschhorn syndrome. So far, eight *NSD2* loss of function variants have been reported in patients from different parts of the world, all were de novo variants.

**Methods:**

In our study, we performed whole exome sequencing for two patients from one family. We also reviewed more *NSD2* mutation cases in pervious literature.

**Results:**

A novel loss of function *NSD2* variant, c.1577dupG (p.Asn527Lysfs*14), was identified in a Chinese family in the proband and her father both affected with intellectual disability. After reviewing more *NSD2* mutation cases in pervious literature, we found none of them had facial features that can be recognized as Wolf-Hirschhorn syndrome. In addition, we have given our proband growth hormone and followed up with this family for 7.5 years.

**Conclusions:**

Here we reported the first familial *NSD2* variant and the long-term effect of growth hormone therapy for patients. Our results suggested *NSD2* mutation might cause a distinct intellectual disability and short stature syndrome.

## Background

Wolf-Hirschhorn syndrome (WHS, MIM 194190) has been regarded as a classic contiguous gene deletion syndrome affecting 1 in 20,000–50,000 live births worldwide, with a 2:1 female/male ratio. Despite its great phenotypic variability, the minimal diagnostic criteria for WHS are defined by typical facial features, prenatal/postnatal growth retardation, developmental delay/intellectual disability (ID) and seizures [1, 2]. Other findings include skeletal anomalies, hypotonia, antibody deficiency, heart defects, hearing loss, urinary tract malformations, structural brain abnormalities, etc. [3]. A 165 kb Wolf-Hirschhorn syndrome critical region (WHSCR) at 4p16.3, which was commonly deleted by all WHS patients, was first described in 1997 by Wright et al. [4]. Within this critical region, the *NSD2* gene, also known as *WHSC1* (*Wolf–Hirschhorn Syndrome Candidate 1* gene), had been recognized as one of the key candidate genes for the contiguous gene deletion syndrome.

**Fig. 1:**
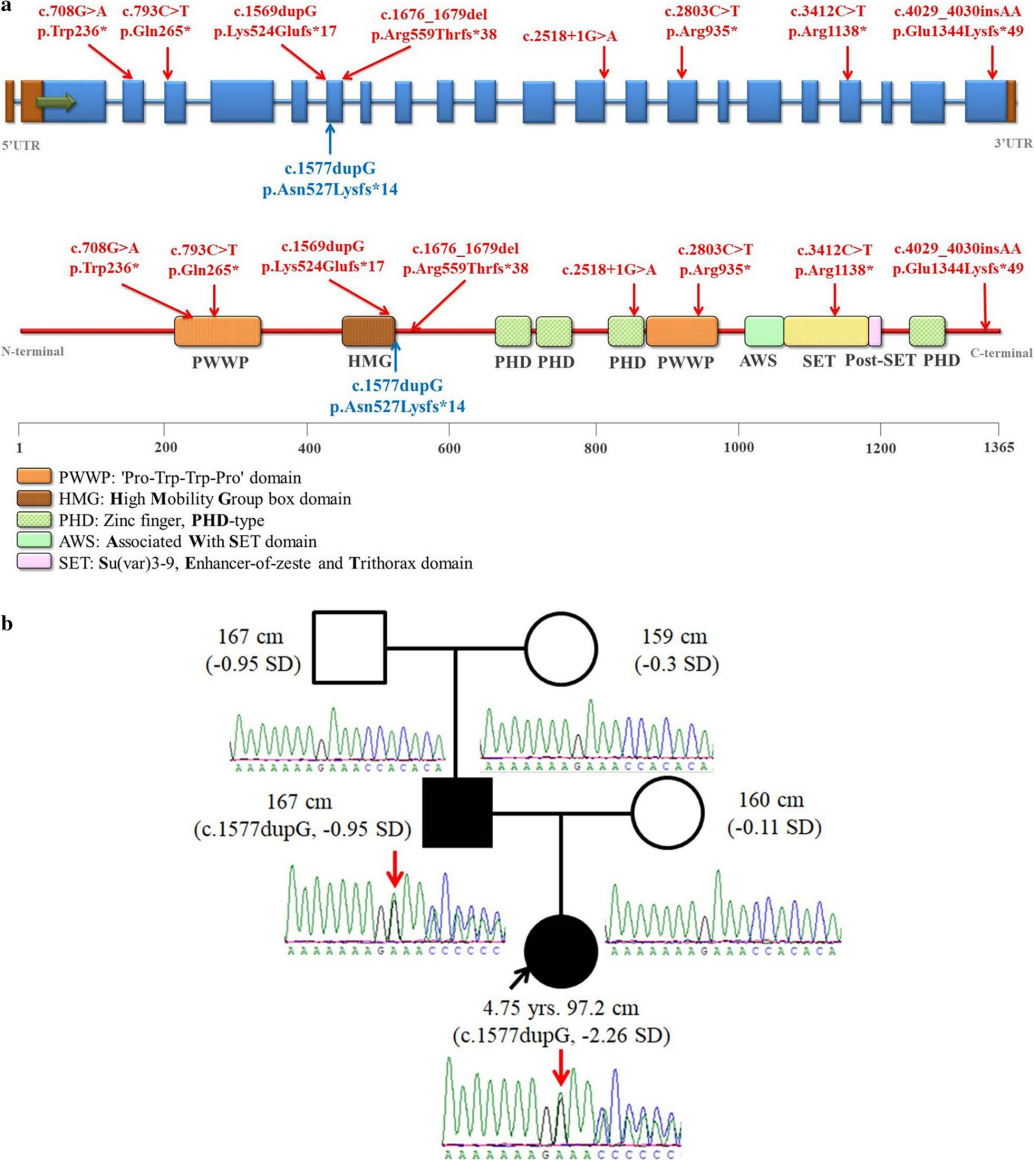
Pathogenic *NSD2* variants. **a**. Distribution of pathogenic variants in the schematic representation of the *NSD2* gene. The red variants (up) are previously reported in patients. The blue variant (down) is the novel variant identified in this study; **b**. Pedigrees and Sanger sequecing of the family with the pathgenic variant

*NSD2* encodes nuclear receptor-binding set domain protein 2, a histone-lysine N-methyltransferase that is believed to play a significant role in normal development [5]. Since 2018, eight patients with loss of function *NSD2* variants have been reported around the world [6–10] (Fig. 1). While most *NSD2* patients presented with overlapping yet atypical features of WHS, thus defining a novel WHS-like disorder, this gene had also been viewed as the disease gene for WHS [9]. Whether or not *NSD2* could account for all WHS phenotypes remain to be further elucidated. Herein, we reported the first familial *NSD2* case from a Chinese family with a novel *NSD2* pathogenic variant. The father had the de novo variant which was passed on to the proband daughter. We describe the clinical phenotypes of both father and daughter. We also evaluated the effectiveness of a long-term growth hormone therapy for the proband based on a 7.5-year follow-up study.

## Methods

### Subjects

The proband was first referred to the Pediatric Endocrine Clinic at Beijing Children’s Hospital due to poor weight gain and growth delay. Written informed consents to participate were obtained from participants and the parents of the participant under the age of 16. The study was approved by the institutional medical ethics committee of Beijing Children’s Hospital.

### Genomic sequencing and variants analysis

DNA was isolated from peripheral blood samples obtained from patients and family members by using the GentraPuregene Blood Kit (QIAGEN, Hilden, Germany) according to the manufacturer’s protocol. The SureSelect Human All Exon Kit (Agilent Technologies, Santa Clara, America) was used for whole exome capture from proband’s DNA. Next generation sequencing was done using Hiseq2000 (Illumina, San Diego, America). Paired-end reads were aligned to the GRCh37/hg19 human reference sequence using Burrows-Wheeler Aligner (BWA) with the MEM algorithm. BAM files were generated by Picard. Sequence reads were recalibrated by RealignerTargetCreator in Genome Analysis Toolkit (GATK) and sequence variants were called by GATK HaplotypeCaller.

Variants were annotated and filtered by Ingenuity Pathway Analysis (https://variants.ingenuity.com). Common variants were filtered based on their frequency in the databases of ExAC (http://exac.broadinstitute.org), gnomAD (V2.11, https://gnomad.broadinstitute.org/) and our internal database. Rare variants were classified following the ACMG/AMP standards and guidelines [11]. All putative pathogenic variants detected by NGS were confirmed by Sanger sequencing. Additional family members were tested for the specific variants. Primer design was performed by Primer Z [12]. Products of PCR amplification (Takara Biotechnology, Co, Ltd., Dalian, China) were purified and sequenced on an ABI PRISM 3730 Genetic Analyzer (Applied Biosystems, Thermo Fisher Scientific, Inc). XHMM software was used to detect CNVs in whole-exome sequencing data as previously described [13].

## Results

### Clinical information

The proband was referred to the clinic at the age of 4 years and 9 months due to poor weight gain and growth retardation since birth. Intrauterine growth retardation was noticed during pregnancy. She was delivered at 33 weeks of gestation by a spontaneous premature labor. Her birth weight was 1900 g (− 3.12SD) and birth length was 41 cm (− 5.11SD). She could sit at 6 months, crawl at 12 months, walk at 15 months independently. She said her first word at 16 months. Her first tooth eruption was at 11 months. After birth, she was consistently shorter than normal children of the same age. Mild developmental delay was noted when she was a toddler. She went to normal primary school but she had a poor academic performance.

At her first visit at the age of 4 years and 9 months, she was 97.2 cm (− 2.26 SD) and 11 kg (− 2.82SD). Her head circumference was 44 cm (<−3SD). Her arm span was 94.5 cm and the upper to lower segment ratio was 1.15:1. She had mild intellectual delay. She presented with a thin and short stature. Her craniofacial features included microcephaly, low hairline, high-arched eyebrows, hypertelorism, epicanthal folds, hypoplastic midface, flat profile, low-set and posterior-rotated ears, relative micrognathia and long neck (Supplemental Table 1). She had normal female external genitalia. Biochemistry tests including blood electrolyte, liver and kidney function, myocardial enzymes, blood fat, thyroid function, cortisol, ACTH, glycosylated hemoglobin, sex hormones were normal. Some endocrine test results are as following: IGF-1142 ng/ml (reference range 49–283), IGF-BP3 3.45 μg/ml (reference range 1.0–4.7), and the peak value of growth hormone stimulation test is (insulin combined with arginine) 10.02 ng/ml. She had normal pituitary MRI. Her karyotype was 46, XX. Electroencephalogram and ultrasonic cardiography were normal. Her bone age was 4 years old. Results of Wechsler Intelligence Scale for Children-IV (WISC-IV) showed an IQ 75.

Her father was 167 cm (− 0.95 SD) and not known to be short as a child, her mother was 160 cm (− 0.11 SD). Her father also had intellectual disability and distinct facial features (Supplemental Table 1). Her father had a seizure after a fever as a child, encephalitis was suspected, but no definite diagnosis was made. Her paternal grandfather was 167 cm (− 0.95 SD) and paternal grandmother was 159 cm (− 0.3 SD), and both had normal intelligence.

For both the proband and the father, the facial features did not prompt a WHS clinical diagnosis even though our patients shared other phenotypes with those previously reported individuals with *NSD2* LOF mutations. Phenotypic features and clinical data of subjects with *NSD2* loss-of-function (LOF) mutation from our report and from patients recently described in scientific literature, are summarized in Table 1 and Supplemental Table 1. Our results proposed it might be a new intellectual disability and short stature syndrome for these patients.

**Table 1:** Common clinical manifestation of *NSD2* truncating mutation cases

Phenotype	Percentage in *NSD2* patients	Percentage in WHS patients
Total number of patients	10	> 300
Intellectual disability/Developmental delay	100% (10/10)	> 75%
Ear abnormal	88% (7/8)	> 75%
Hypertelorism	86% (6/7)	> 75%
High-arched eyebrows	86% (6/7)	> 75%
Wide nasal bridge	86% (6/7)	> 75%
Abnormal teeth^a^	86% (6/7)	50–75%
Hypotonia	80% (8/10)	> 75%
Intrauterine/postnatal growth retardation	80% (8/10)	> 75%
Feeding difficulties	78% (7/9)	> 75%
Microcephaly^a^	60% (6/10)	> 75%
Micrognathia^a^	57% (4/7)	> 75%
Epicanthal folds^a^	57% (4/7)	> 75%
Downturned corners of mouth^a^	57% (4/7)	> 75%
Skeletal anomalies	50% (3/6)	50–75%
Short philtrum^a^	43% (3/7)	> 75%
Stereotypies (hand washing/flapping, rocking)	33% (2/6)	25–50%
Prominent glabella^a^	29% (2/7)	> 75%
Craniofacial asymmetry^a^	29% (2/7)	50–75%
High forehead^a^	25% (2/8)	> 75%
Hearing loss	25% (1/4)	25–50%
Skin changes (hemangioma; marble/dry skin)^a^	20% (1/5)	50–75%
Genitourinary tract anomalies^a^	17% (1/6)	25–50%
Gut anomalies	17% (1/6)	< 25%
Esophagus anomalies	17% (1/6)	< 25%
Structural brain anomalies^a^	14% (1/7)	25–50%
Liver anomalies	14% (1/7)	< 25%
Seizures and/or distinctive Electroencephalogram abnormalities^a^	11% (1/9)	> 75%

*WHS* Wolf-Hirschhorn syndrome, *HGVS* Human genome variation society, *NA* Not Available

^a^features with different incidence rates in *NSD2* and WHS patients

### Genetic findings

We identified a novel frameshift variant (NM_001042424: c.1577dupG, p.Asn527Lysfs*14) in *NSD2* in both proband and her affected father, this variant was absent from paternal grandfather and grandmother, Fig. 1). Thus it is a de novo variant for the father (Strong evidence of pathogenicity 2). This variant is located next to the C-terminal of High Mobility Group (HMG) box domain and is predicted to lead to nonsense-mediated mRNA decay (NMD) (Very strong evidence of pathogenicity 1, Fig. 1). This variant was previously unreported and absent from databases (Moderate evidence of pathogenicity 2). This is a pathogenic variant following ACMG/AMP classification guideline (1 Very Strong + 1 Strong + 1 Moderate). No pathogenic or likely pathogenic CNV were identified based on WES data using XHMM software.

### Growth hormone treatment and outcome

**Fig. 2:**
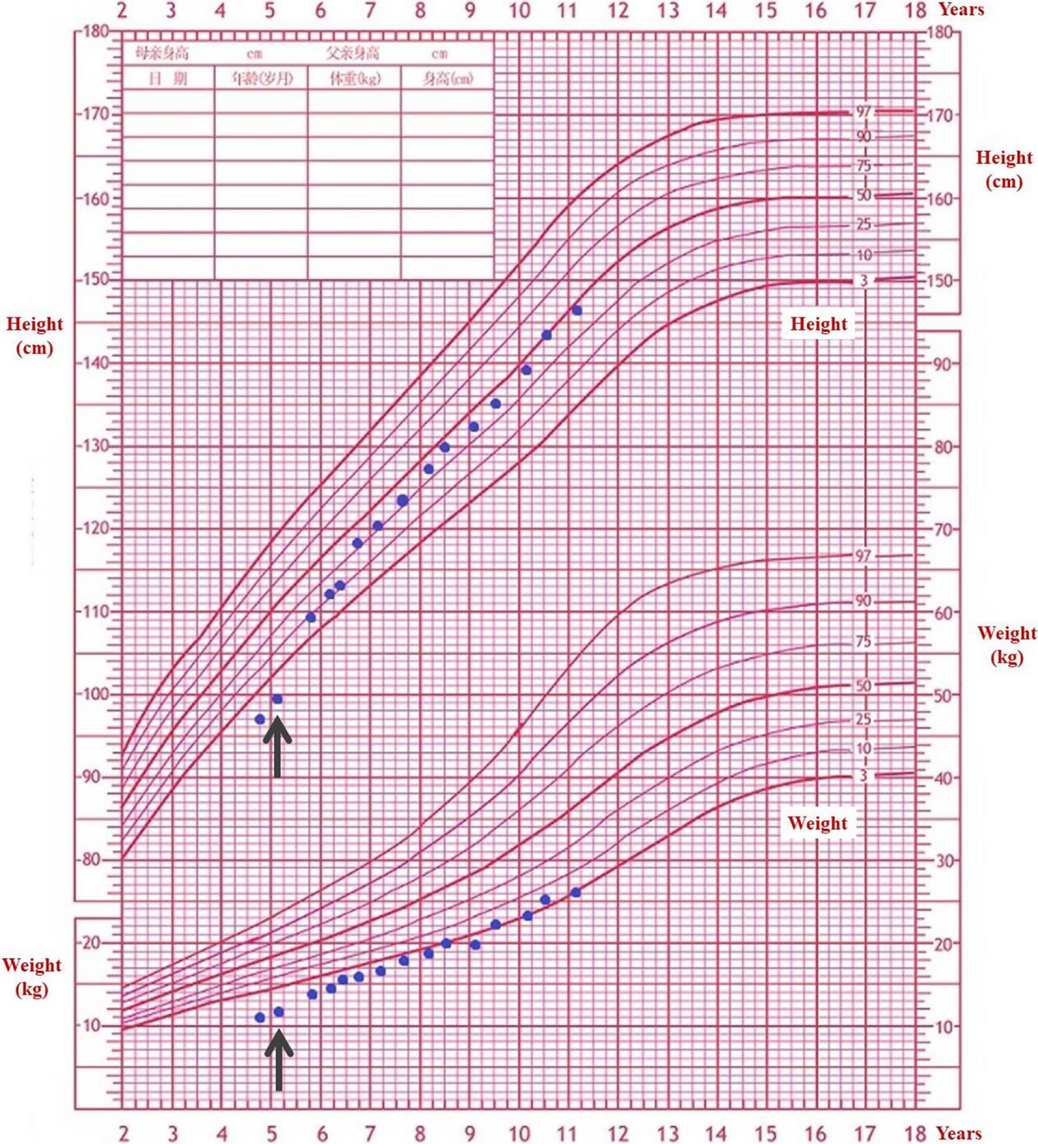
Growth chart of the proband. The arrows indexed the initiation of growth hormone therapy

GH therapy was initiated when she was 5 years and 2 months on the basis of short stature. Her bone age was 5 years old then. The height velocity was 5.52 cm/year before treatment. The first year height velocity after treatment was 12.5 cm/year. She was compliant with GH injections (0.15–0.17 IU/kg/day Q. N, Subcutaneous injection), and there were no complications. The growth curve is shown in Fig. 2 and Supplemental Table 2. During GH therapy, all the monitoring indexes were normal, including biochemistry, thyroid function test, IGF-1, insulin, sex hormones (including luteinizing hormone, follicle stimulating hormone, estradiol, testosterone, progesterone, prolactin), blood routine, urine routine, HbA1C, Electroencephalogram and bone age. The proband had normal puberty when she was 10.5 years of age (breast Tanner II), and her age of menarche was 12.7 years old. The outcome of growth hormone treatment is good (Height SDS increased from − 2.25 SD to − 0.03 SD).

## Discussion

WHS is a rare intellectual disability syndrome affecting 1 in 20,000–50,000 births. The phenotypes of WHS patients are variable and correlate with 4p16 deletion sizes: Patients with small deletions (< 3.5 Mb) often had mild WHS, patients with a deletion of 5–18 Mb has classical WHS and patients with large deletion (> 22–25 Mb) have severe phenotypes [2]. Yet all patients had typical facial dysmorphisms comprised by prominent forehead and glabella, highly arched eyebrows, hypertelorism, epicanthal folds, short philtrum, distinct mouth, and micrognathia [1, 14]. Patients with deletions involving the 165 kb WHS critical region usually exhibit these typical features of WHS [4], but individuals with LOF mutations of *NSD2* often do not present with the full WHS facial features [1, 3, 9]. They exhibited milder or less recognizable facial dysmorphism comparing to typical 4p16.3 deletion WHS patients [6, 8–10, 15]. As the phenotypes of *NSD2* patients did not meet the minimal diagnostic criteria for WHS [1, 14], a new intellectual disability and short stature syndrome has been proposed for these patients [15]. The major features (> 50%) of this syndrome included intellectual disability/developmental delay, ear abnormal, hypertelorism, high-arched eyebrows, wide nasal bridge, abnormal teeth, hypotonia, intrauterine/postnatal growth retardation, feeding difficulties, microcephaly, micrognathia, epicanthal folds, downturned corners of mouth and skeletal anomalies. Other minor (25–50%) features included short philtrum, prominent glabella, craniofacial asymmetry, high forehead, strabismus, hearing loss and stereotypies (Table 1, Supplemental Table 1). Our results provided basis for the clinical diagnosis of *NSD2* patients in the future. Due to the lack of recognizable facial features of WHS in both of our patients, our findings support the notion that *NSD2* mutation is not fully responsible for all features of WHS, especially the facial features (e.g. ocular anomalies and cleft lip/palate). Other neighboring genes beyond *NSD2* mutation, as *FGFRL1* could be responsible for the development of dysmorphology [16, 17].

Growth retardation was reported in 80% of WHS [1] and *NSD2* patients (Supplemental Table 1) but short stature in WHS is not usually linked to growth hormone deficiency, thus GH supplementation is not often used for WHS patients. So far only six WHS patients underwent GH therapy [18–22] and five of them had detailed information which demonstrated a significant increase in linear height during childhood in all these patients, the longest follow-up of the treatment was 7.5 years [19]. The annual height gain ranged from 0.37 SDS/year to 0.97 SDS/year. The results of these patients showed significant increase in linear height during childhood. (Supplemental Table 3). Currently, none of *NSD2* patients had been tested for growth hormone and none were treated with GH. The proband had normal GH stimulation test result but her height and weight were consistently lower than 3rd percentile before treatment (Fig. 2). Our proband is the first *NSD2* patient underwent GH therapy. After a 7.5-year treatment, her height increased from − 2.25 SD to − 0.03 SD (Fig. 2 and Supplemental Table 2). No obvious negative effects were observed and the bone age of our patient was continuously consistent with her chronological age. This study provided first case evidence for *NSD2* mutation patients to improve their height with GH therapy. It was notable that all the other reported variants in pervious literature were de novo, and our research reported the first familial *NSD2* variant. In our results, even family members with the same variant had variable phenotypes (for example, the father did not exhibit short stature), indicating an incomplete penetrance on specific features. Different genetic background among family members might one of the explanations. However, more cases are needed to further demonstrate this point.

## Conclusions

In summary, we reported the first familial *NSD2* cases with a novel loss of function variant. Both patients presented with main features reported in previous *NSD2* patients who presented with overlapping features of WHS. But the lack of typical facial features in our patients and in other *NDS2* patients suggested that *NSD2* gene mutation along is not sufficient to account for all WHS phenotypes. Other neighboring genes should be playing roles for the manifestation of typical facial features. The search should continue to evaluate the involvement of other genes in WHS. Our study also summarized the phenotype of *NSD2* patients and provided basis for the clinical diagnosis in the future. *NSD2* patients who had short stature can benefit from GH treatment even though GH was not deficient. The long-term GH treatment appears to be effective and safe. More cases will be needed to fully evaluate the benefit of GH treatment and define a potential novel syndrome.

## Supplementary Information

**Additional file 1: Table S1.** Clinical manifestation comparison in WHS patients and *NSD2* truncating mutation cases.

**Additional file 2: Table S2.** GH therapy outcome of the proband.

**Additional file 3: Table S3.** Growth hormone therapy of previously repoerted Wolf-Hirschhorn syndrome cases.

## Data Availability

The dataset used and/or analyzed during the current study are available from the corresponding author on reasonable request. Human genome version hg19/GRCh37 is available from the UCSC Genome Browser website (genome.ucsc.edu). Sequencing files are available from the NCBI BioProject database (www.ncbi.nlm.nih.gov/bioproject/679686) and Mendeley Data (data.mendeley.com/datasets/vckjp394p4/1).

## Abbreviations

WHS: Wolf-Hirschhorn syndrome
NSD2: Nuclear receptor-binding set domain protein 2 gene
GH: Growth hormone
ID: Intellectual disability
WHSCR: Wolf-Hirschhorn syndrome critical region
WHSC1: Wolf–Hirschhorn Syndrome Candidate 1 gene
BWA: Burrows-Wheeler Aligner
GATK: Genome Analysis Toolkit
WISC-IV: Wechsler Intelligence Scale for Children-IV
LOF: Loss-of-function
NMD: Nonsense-mediated mRNA decay
HMG: High Mobility Group

## References

[1] Battaglia A, Carey JC, South ST (2015). Wolf-Hirschhorn syndrome: a review and update. Am J Med Genet C.

[2] Battaglia A, Filippi T, Carey JC (2008). Update on the clinical features and natural history of wolf-Hirschhorn (4p-) syndrome: experience with 87 patients and recommendations for routine health supervision. Am J Med Genet C.

[3] Battaglia A, Carey JC, South ST, Adam MP, Ardinger HH, Pagon RA, Wallace SE, LJH B, Stephens K (1993). Wolf-Hirschhorn syndrome. GeneReviews ((R)). Seattle (WA).

[4] Wright TJ, Ricke DO, Denison K, Abmayr S, Cotter PD, Hirschhorn K (1997). A transcript map of the newly defined 165 kb wolf-Hirschhorn syndrome critical region. Hum Mol Genet.

[5] Kim JY, Kee HJ, Choe NW, Kim SM, Eom GH, Baek HJ (2008). Multiple-myeloma-related WHSC1/MMSET isoform RE-IIBP is a histone methyltransferase with transcriptional repression activity. Mol Cell Biol.

[6] Jiang Y, Sun H, Lin Q, Wang Z, Wang G, Wang J (2019). De novo truncating variant in NSD2gene leading to atypical wolf-Hirschhorn syndrome phenotype. BMC Med Genet.

[7] Barrie ES, Alfaro MP, Pfau RB, Goff MJ, McBride KL, Manickam K (2019). De novo loss-of-function variants in NSD2 (WHSC1) associate with a subset of wolf-Hirschhorn syndrome. Cold Spring Harb Mol Case Stud.

[8] Lozier ER, Konovalov FA, Kanivets IV, Pyankov DV, Koshkin PA, Baleva LS (2018). De novo nonsense mutation in WHSC1 (NSD2) in patient with intellectual disability and dysmorphic features. J Hum Genet.

[9] Derar N, Al-Hassnan ZN, Al-Owain M, Monies D, Abouelhoda M, Meyer BF (2018). De novo truncating variants in WHSC1 recapitulate the wolf-Hirschhorn (4p16.3 microdeletion) syndrome phenotype. Genet Med.

[10] Boczek NJ, Lahner CA, Nguyen TM, Ferber MJ, Hasadsri L, Thorland EC (2018). Developmental delay and failure to thrive associated with a loss-of-function variant in WHSC1 (NSD2). Am J Med Genet A.

[11] Richards S, Aziz N, Bale S, Bick D, Das S, Gastier-Foster J (2015). Standards and guidelines for the interpretation of sequence variants: a joint consensus recommendation of the American College of Medical Genetics and Genomics and the Association for Molecular Pathology. Genet Med.

[12] Tsai MF, Lin YJ, Cheng YC, Lee KH, Huang CC, Chen YT (2007). PrimerZ: streamlined primer design for promoters, exons and human SNPs. Nucleic Acids Res.

[13] Fromer M, Purcell SM (2014). Using XHMM software to detect copy number variation in whole-exome sequencing data. Curr Protoc Hum Genet.

[14] Zollino M, Murdolo M, Marangi G, Pecile V, Galasso C, Mazzanti L (2008). On the nosology and pathogenesis of wolf-Hirschhorn syndrome: genotype-phenotype correlation analysis of 80 patients and literature review. Am J Med Genet C: Semin Med Genet.

[15] Zollino M, Doronzio PN (2018). Dissecting the wolf-Hirschhorn syndrome phenotype: WHSC1 is a neurodevelopmental gene contributing to growth delay, intellectual disability, and to the facial dysmorphism. J Hum Genet.

[16] Catela C, Bilbao-Cortes D, Slonimsky E, Kratsios P, Rosenthal N, Te Welscher P (2009). Multiple congenital malformations of wolf-Hirschhorn syndrome are recapitulated in Fgfrl1 null mice. Dis Model Mech.

[17] Engbers H, van der Smagt JJ, van’t Slot R, Vermeesch JR, Hochstenbach R, Poot M (2009). Wolf-Hirschhorn syndrome facial dysmorphic features in a patient with a terminal 4p16.3 deletion telomeric to the WHSCR and WHSCR 2 regions. Eur J Hum Genet.

[18] Austin DE, Gunn AJ, Jefferies CA (2015). Severe short stature and wolf-Hirschhorn syndrome: response to growth hormone in two cases without growth hormone deficiency. Oxf Med Case Reports.

[19] Siew JX, Yap F (2018). Growth trajectory and pubertal tempo from birth till final height in a girl with wolf-Hirschhorn syndrome. Endocrinol Diabetes Metab Case Rep.

[20] Andersen EF, Carey JC, Earl DL, Corzo D, Suttie M, Hammond P (2014). Deletions involving genes WHSC1 and LETM1 may be necessary, but are not sufficient to cause wolf-Hirschhorn syndrome. Eur J Hum Genet.

[21] Lindeman-Kusse MC, Van Haeringen A, Hoorweg-Nijman JJ, Brunner HG (1996). Cytogenetic abnormalities in two new patients with Pitt-Rogers-Danks phenotype. Am J Med Genet.

[22] Van Buggenhout G, Melotte C, Dutta B, Froyen G, Van Hummelen P, Marynen P (2004). Mild wolf-Hirschhorn syndrome: micro-array CGH analysis of atypical 4p16.3 deletions enables refinement of the genotype-phenotype map. J Med Genet.

